# *N*-Ribosyltransferase From *Archaeoglobus veneficus*: A Novel Halotolerant and Thermostable Biocatalyst for the Synthesis of Purine Ribonucleoside Analogs

**DOI:** 10.3389/fbioe.2020.00593

**Published:** 2020-06-16

**Authors:** Javier Acosta, Jon Del Arco, Victor Pisabarro, Federico Gago, Jesús Fernández-Lucas

**Affiliations:** ^1^Applied Biotechnology Group, Universidad Europea de Madrid, Urbanización El Bosque, Madrid, Spain; ^2^Department of Biomedical Sciences and “IQM-CSIC Associated Unit”, School of Medicine and Health Sciences University of Alcalá, Madrid, Spain; ^3^Grupo de Investigación en Ciencias Naturales y Exactas, GICNEX, Universidad de la Costa, CUC, Barranquilla, Colombia

**Keywords:** nucleosides, extremophiles, nucleoside 2′-deoxyribosyltransferase, transglycosylation, homology modeling

## Abstract

Nucleoside-2′-deoxyribosyl-transferases (NDTs) catalyze a transglycosylation reaction consisting of the exchange of the 2′-deoxyribose moiety between a purine and/or pyrimidine nucleoside and a purine and/or pyrimidine base. Because NDTs are highly specific for 2′-deoxyribonucleosides they generally display poor activity on modified C2′ and C3′ nucleosides and this limitation hampers their applicability as biocatalysts for the synthesis of modified nucleosides. We now report the production and purification of a novel NDT from *Archaeoglobus veneficus* that is endowed with native ribosyltransferase activity and hence it is more properly classified as an *N*-ribosyltransferase (*Av*NRT). Biophysical and biochemical characterization revealed that *Av*NRT is a homotetramer that displays maximum activity at 80°C and pH 6 and shows remarkably high stability at high temperatures (60–80°C). In addition, the activity of *Av*NRT was found to increase up to 2-fold in 4 M NaCl aqueous solution and to be retained in the presence of several water-miscible organic solvents. For completeness, and as a proof of concept for possible industrial applications, this thermophilic and halotolerant biocatalyst was successfully employed in the synthesis of different purine ribonucleoside analogs.

## Introduction

Nucleoside analogs (NAs) comprise a group of synthetic molecules that mimic natural nucleosides and have been widely used as anticancer agents and to treat some viral and fungal infections since the late 1960's (De Clercq, 2005; Parker, 2009; Jordheim et al., 2013). More recently, NAs are also receiving increased attention because of their potential to be repurposed to fight bacterial drug resistance (Thomson and Lamont, 2019).

Nowadays, the biocatalytic synthesis of NAs is gaining momentum over the traditional multistep and more environmentally harmful chemical methods (Lewkowicz and Iribarren, 2017; Fernández-Lucas and Camarasa, 2019). Enzyme-catalyzed synthesis by whole cells or purified proteins offers several advantages, such as one-pot reactions, controlled stereo-, regio-, and enantiospecificities, and mild reaction conditions. In this regard, the most commonly used synthetic route for the synthesis of NAs is the transglycosylation reaction catalyzed by nucleoside phosphorylases or 2′-deoxyribosyltransferases (Fresco-Taboada et al., 2013; Lapponi et al., 2016; Del Arco and Fernández-Lucas, 2018; Kamel et al., 2019a; Lewkowicz and Iribarren, 2019; Trelles et al., 2019).

Nucleoside-2′-deoxyribosyltransferases (NDTs) are the most representative members of the *N*-deoxyribosyltransferase family (NDT family) which belongs to the *N*-(deoxy)ribosyltransferase-like superfamily (PFAM entry CL0498), also including ADP-ribosyl cyclase-like families. NDTs catalyze the interchange of the 2′-deoxyribose moiety between a purine and/or pyrimidine 2′-deoxynucleoside (donor) and a purine and/or pyrimidine base (acceptor) (Kaminski, 2002; Fernández-Lucas et al., 2010; Fresco-Taboada et al., 2013; Crespo et al., 2017; Del Arco et al., 2019a). According to their substrate specificity, NDTs are usually classified as: (i) type I NDTs (PDT), which perform this type of transglycosylation reaction between purine bases (Fresco-Taboada et al., 2013; Pérez et al., 2018) and (ii) type II NDTs (NDT), which do not discriminate between purines and pyrimidines (Kaminski, 2002; Fernández-Lucas et al., 2010; Fresco-Taboada et al., 2016). Interestingly, NDTs are highly regioselective for natural bases and a high number of nucleobase derivatives (Fresco-Taboada et al., 2013; Acosta et al., 2018). However, several examples of NDT-mediated glycosylation at multiple sites have been recently reported for several size-expanded purines (Ye et al., 2014) and azole derivatives (Vichier-Guerre et al., 2017).

Despite their great potential for performing important chemical transformations, the use of NDTs as industrial biocatalysts for the synthesis of nucleoside analogs is often hindered by their strict preference for 2′-deoxyribonucleosides. In this respect, several attempts have been made for tailoring NDT active sites toward new substrate specificities on the ribose moiety (Kaminski et al., 2008; Kaminski and Labesse, 2013; Del Arco et al., 2019b; Li et al., 2019). Additionally, most of the reported NDTs up to date are mesophilic enzymes, with the exception of NDTs from cold-adapted *Bacillus psychrosaccharolyticus* (Fresco-Taboada et al., 2014) and the extremophilic cyanobacteria *Chroococcidiopsis thermalis* PCC 7203 (*Ct*NDT) (Del Arco et al., 2018a). Consequently, mesophilic enzymes usually display low stability and short lifespan under the harsh conditions usually required for industrial processes, such as extreme pH values, high temperatures or the presence of organic solvents. The use of enzymes from microorganisms naturally living in extreme environments has proved to be a successful approach to circumventing the abovementioned drawbacks associated to industrial implementation. More specifically, the choice of enzymes from thermophiles for the synthesis of nucleosides and nucleotides offers, among other advantages, the possibility of using high-temperature reactions, resulting in higher substrate solubilization and increased overall reaction rates (Almendros et al., 2012; Del Arco et al., 2017, 2018a,b, 2019c; Atalah et al., 2019; Kamel et al., 2019b; Zhou et al., 2019).

Another interesting source of “extremozymes” (Elleuche et al., 2014) are halophilic organisms (De Lourdes Moreno et al., 2013). Halophilic enzymes (commonly named haloenzymes) are usually active and stable at high salt concentrations, but sometimes also withstand extreme high temperatures and pH values, as well as the presence of organic solvents and other chaotropic agents. Because of this, halophiles are a promising source of useful enzymes for industry nowadays.

Herein we report the expression, purification and the biochemical characterization of a novel putative NDT enzyme naturally encoded in the motile archaeal coccus *Archaeloglobus veneficus*. This enzyme appears as the first wild-type NDT to act on ribonucleosides and therefore we think it is more appropriately classified as an *N*-ribosyltransferase (NRT). Interestingly, *Av*NRT is a homotetramer with remarkable activity and stability at extremely high temperatures, and also shows a significant ionic strength dependence. For completeness, the enzymatic production of different modified purine nucleosides was carried out to assay the potential of *Av*NRT as an industrial biocatalyst for the synthesis of nucleoside analogs.

## Materials and Methods

### Materials and Chemicals

Cell culture medium reagents were purchased from Difco (St. Louis, USA). Triethyl ammonium acetate buffer was provided by Sigma-Aldrich (Madrid, Spain). All other reagents and organic solvents were purchased from Symta (Madrid, Spain). Nucleosides and nucleobases were provided by Carbosynth Ltd., (Compton, UK).

### Cloning, Expression and Protein Purification

After exhaustive *in silico* mining of unusual NDT candidates, we found a gene which encodes a putative NDT from *Archaeoglobus veneficus* SNP6 (European Nucleotide Archive code: AEA46496.1; UniProtKB code F2KPY9). The corresponding *nrt* gene was purchased from GenScript (USA) as a *Nde*I-*Eco*RI fragment subcloned into the expression vector pET28b(+). The N-terminal His_6_-tagged *Av*NRT was expressed by growing the *E. coli* BL21(DE3)_pET28bAv*NRT*_ transformed cells in Luria Bertani medium supplemented with kanamycin 50 μg/mL. Protein overexpression was induced by adding 0.5 mM isopropyl-β-D-1-thiogalactopyranoside (IPTG) to exponential cultures and the cells were further grown for 3 h. Cells were harvested by centrifugation at 3,600 × *g*, the resulting pellet was resuspended in 10 mM sodium phosphate buffer pH 7, and cellular disruption was performed using a digital sonifier. The lysates were centrifuged at 16,500 × *g* for 30 min at 4°C, the supernatant was heated at 70°C for 20 min, and insoluble material was removed by centrifugation. The cleared lysates were then loaded onto a 5-mL HisTrap FF column (GE Healthcare), pre-equilibrated in a binding buffer (20 mM Tris-HCl buffer, pH 7.5, with 100 mM NaCl and 20 mM imidazole). Bound proteins were eluted using a linear gradient of imidazole (from 20 to 500 mM). Fractions containing *Av*NRT were identified by SDS-PAGE, pooled, concentrated and loaded onto a HiLoad 16/60 Superdex 200 prep grade column (GE Healthcare) pre-equilibrated in 20 mM sodium phosphate, pH 7. Fractions containing *Av*NRT were identified by SDS-PAGE. Protein concentration was determined spectrophotometrically by UV absorption 280 nm using a ε_280_ = 9,970 M^−1^cm^−1^.

### Analytical Ultracentrifugation Analysis

Sedimentation velocity experiments for *Av*NRT were carried out in 20 mM Tris-HCl (pH 8, 20 °C, 50,000 × *g*) using an Optima XL-I analytical ultracentrifuge (Beckman-Coulter Inc.) equipped with UV-VIS absorbance and Raleigh interference detection systems, an An-60Ti rotor and standard (12 mm optical path) double-sector center pieces of Epon-charcoal. Sedimentation profiles were recorded at 292 nm. Sedimentation coefficient distributions were calculated by least-squares boundary modeling of sedimentation velocity using the continuous distribution *c(s)* Lamm equation model as implemented by SEDFIT 14.7 g.

Baseline offsets were measured afterwards at 200,000 × *g*. The apparent sedimentation coefficient distribution, *c*(*s*), and sedimentation coefficients were calculated from the sedimentation velocity data using SEDFIT (Brown and Schuck, 2006). The experimental sedimentation coefficients were corrected to standard conditions (water, 20°C, and infinite dilution) using SEDNTERP software to obtain the corresponding standard values (*s*_20_,_*w*_).

### Enzyme Activity Assay

The standard activity assay (enzymatic hydrolysis of ribo- or 2′-deoxyribonucleosides) was performed by incubating 10–50 μL of free extracts or 11.2–22.4 μg of pure enzyme with a 40 μL solution containing 1 mM ribo- or 2′-deoxyribonucleoside in 50 mM sodium phosphate buffer, pH 6. The reaction mixture was incubated at 80 °C for 20 min (300 rpm). The enzyme was inactivated by adding 40 μL of cold methanol in an ice bath and heating for 5 min at 100 °C. After centrifugation at 9,000 × g for 10 min, the samples were half-diluted with water and nucleobase production was analyzed and quantitatively measured using HPLC. Under such conditions, one activity unit (U) was defined as the amount of enzyme (mg) producing 1 μmol/min (IU) of resulting nucleobase from corresponding nucleoside under the assay conditions. All determinations were carried out in triplicate and the maximum error was <5%. The statistical dispersion of the replicates was determined according to standard deviation analysis.

### Influence of pH, Temperature and Ionic Strength on Enzyme Activity

In order to stablish the optimal operational conditions, we assayed the effect of different reaction parameters on enzyme activity, such as pH, T and ionic strength. The optimum pH of *Av*NRT was initially determined according to the standard activity assay using guanosine (Guo) as substrate, and sodium citrate (pH 4–6), MES buffer (pH 5.5–7) or sodium phosphate (pH 6–8.5) as reaction buffers (50 mM). The optimum temperature was determined using the standard assay over a 40–100°C temperature range. In a similar way, the effect of ionic strength on enzyme activity was measured at different NaCl concentrations ranging from 0 to 4 M.

### Thermal Stability

In order to assess optimal storage conditions, *Av*NRT was kept at 4 and−80°C in 10 mM sodium phosphate buffer (pH 7) for 365 days. Periodically, samples were taken, and the enzymatic hydrolysis of guanosine was evaluated. Storage stability was defined as the relative activity between the first and successive reactions. Moreover, the thermal stability was further studied by incubating 22.4 μg of pure enzyme in 10 mM sodium phosphate buffer, pH 7, for 24 h at different temperatures (60–80°C).

### Influence of Water-Miscible Solvents on Enzyme Activity

To determine the enzymatic activity in non-conventional media, the enzymatic hydrolysis of guanosine was assayed in the presence of different protic and aprotic organic solvents. To this end, 11.2 μg of pure enzyme were added to a 40 μL solution containing 1 mM guanosine in 50 mM sodium phosphate buffer pH 6, in the presence of 20% (v/v) polar protic (MeOH, EtOH, isopropanol, glycerol, ethylene glycol, and propylene glycol) and aprotic co-solvents (acetonitrile, acetone, chloroform, *N, N*-dimethylformamide, DMSO and ethyl acetate). The reaction mixture was incubated at 80°C for 20 min (300 rpm).

### Enzymatic Synthesis of Nucleosides

To assess the transglycosylation activity of *Av*NRT, 22.4 μg of pure enzyme were incubated in a reaction mixture containing 0.75 mM guanosine or 2′-deoxyguanosine and 0.75 mM purine nucleobase (2,6-diaminopurine, 6-methoxyguanine, adenine, 2-fluoroadenine and 2-chloroadenine), in 50 mM sodium phosphate buffer, pH 6, NaCl 4 M. The reaction mixtures were incubated at 80°C and 300 rpm in an orbital shaker for different reaction times (10–120 min).

### Bioinformatics Analysis, Homology Modeling and Molecular Dynamics Simulations

The pairwise amino acid sequence alignment was performed with BLASTP (http://blast.ncbi.nlm.nih.gov/Blast.cgi), using UniProtKB and PDB as reference databases. Multiple sequence alignment of amino acid sequences from NDTs and (d)NMP glycosidases was carried out using CLUSTALΩ (https://www.ebi.ac.uk/Tools/msa/clustalo/). The resulting figure was generated by the ESPript server 3.0. (http://espript.ibcp.fr/ESPript/ESPript/). Several 3D structural models of an *Av*NRT homodimer were built using the threading methods implemented in the Phyre 2.0 (Kelley et al., 2015) and Swiss-Model (Biasini et al., 2014) servers by providing the amino acid sequence deposited in UniProtKB under the code F2KPY9. The best protein templates of known 3D structure were the dimeric PDTs from *Leishmania mexicana* and *Trypanosoma brucei*, and the tetrameric putative NDT from *Enterococcus faecalis* v583 (PDB entries 6QAI, 2A0K, and 3EHD, respectively). None of these models by itself, however, provided full consistency and we found it necessary to make a composite using the information derived from the multiple sequence alignment performed by the Dali server (Holm and Laakso, 2016) on NDT structural neighbors (Figure S1) and previously employed for the modeling of *Ct*NDT (Del Arco et al., 2018a). The RCD+ server (López-Blanco et al., 2016) was employed to generate alternative loop conformations of the intrinsically disordered regions. The AMBER force field (Salomon-Ferrer et al., 2013) was used for progressive energy refinement in explicit solvent followed by molecular dynamics and simulated annealing according to a previously described protocol (Del Arco et al., 2019b). PyMOL was used for structure visualization, molecular editing, and figure preparation (Delano, 2002).

### Analytical Methods

Nucleoside production was analyzed quantitatively with an ACE 5-μm C18-PFP 250 mm × 46 mm column (Advanced Chromatography Technologies) pre-equilibrated in 100% triethyl ammonium acetate. Elution was carried out by a discontinuous gradient, 0–13 min, 100 to 90% triethyl ammonium acetate and 0 to 10% acetonitrile, and 13–20 min, 90 to 100% triethyl ammonium acetate and 10 to 0% acetonitrile. Retention times for the reference nucleobases and nucleosides (hereafter abbreviated according to the recommendations of the IUPAC-IUB Commission on Biochemical Nomenclature) were as follows: 2,6-diaminopurine (2,6-DAP), 10.1 min; 2,6-diaminopurine-2′-deoxyribose (2,6-DAPdRib), 13.5 min; 2,6-diaminopurine riboside (2,6-DAPRib), 2-fluoroadenine (2-FAde), 10.5 min; 2′-deoxy-2-fluoroadenosine (dFAdo), 15.2 min; 2-fluoroadenosine (FAdo), 13.2 min; 2-chloroadenine (2-ClAde), 12.3 min; 2′-deoxy-2-chloroadenosine (dClAdo), 16.2 min; 2-chloroadenosine (ClAdo), 14.6 min; 6-methoxyguanine (6-MeOGua), 11.7 min; 6-methoxy-2′-deoxyguanosine (6-MeOdGuo), 16.8 min; 6-methoxyguanosine (6-MeOGuo), 14.7 min; adenine (Ade), 10.2 min; 2′-deoxyadenosine (dAdo), 14.5 min; adenosine (Ado), 13.8 min; cytosine (Cyt), 3.9 min; 2′-deoxycytidine (dCyd), 7.6 min; guanine (Gua), 7.1 min; 2′-deoxyguanosine (dGuo), 11.0 min; guanosine (Guo), 10.2 min; hypoxanthine (Hyp), 6.8 min; 2′-deoxyinosine (dIno), 10.1; inosine (Ino), 9.2; uracil (Ura), 4.2 min; 2′-deoxyuridine (dUri), 8.2 min; uridine (Uri), 7.0 min. To confirm the reaction products, commercial nucleoside analogs were used as HPLC standards.

## Results and Discussion

### Bioinformatics Analysis of *AvNRT*

**Figure 1:**
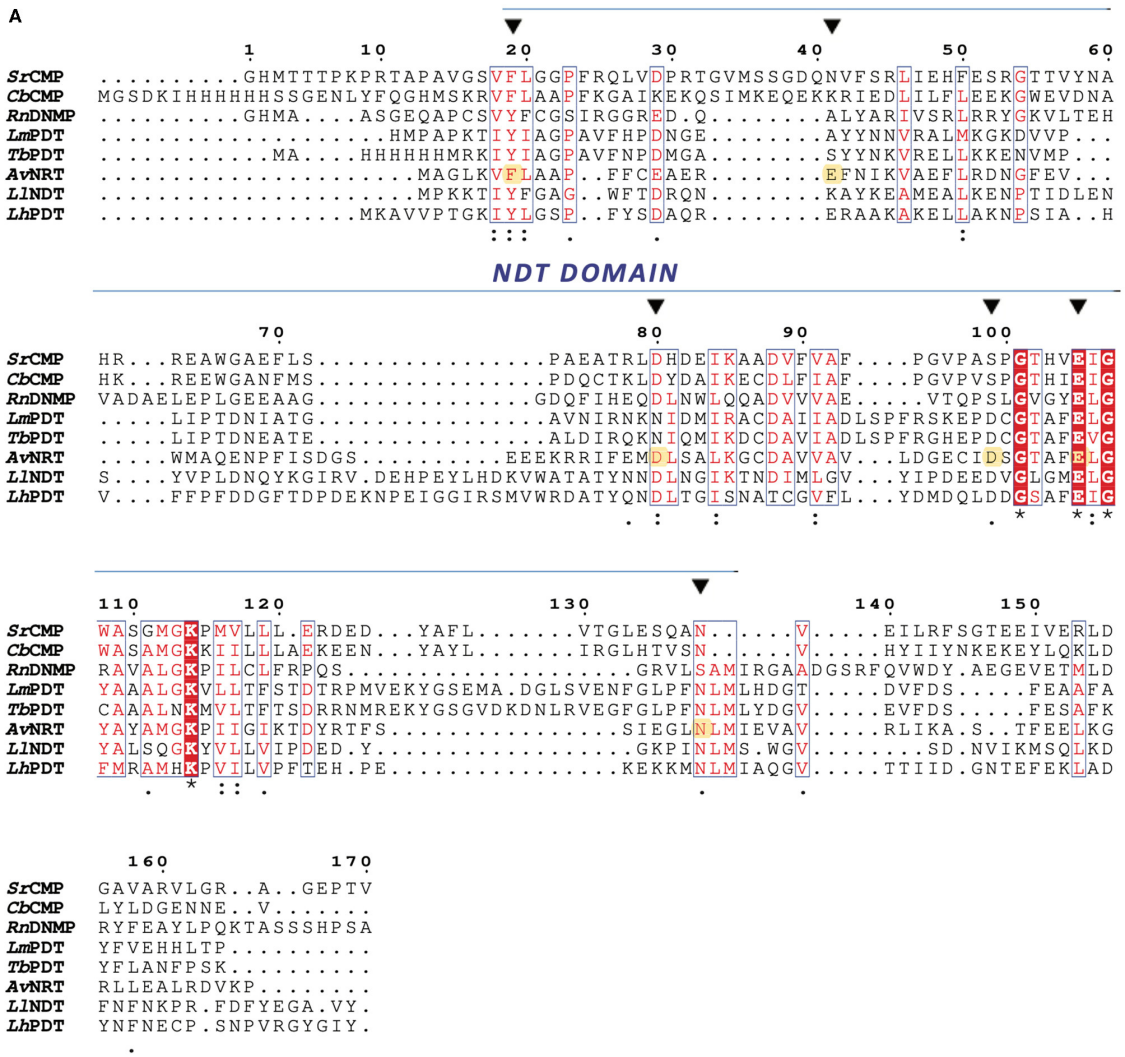
Multiple sequence alignment of type I NDTs (PDTs) from *Lactobacillus helveticus* (*Lh*PDT), *Trypanosoma brucei* (*Tb*PDT), and *Leishmania mexicana* (*Lm*PDT), a type II NDT from *Lactobacillus leichmannii* (*Ll*NDT), and NMP glycosidases from *Streptomyces rimofaciens* (*Sr*CMP, MilB), *Clostridium botulinum* (*Cb*CMP, BcmB), *Rattus norvegicus* (*Rn*DNMP, Rcl). Amino acids for each polypeptide sequence were independently numbered. Active site residues are indicated by an inverted black triangle (▼) and highlighted in yellow in the *Av*NRT amino acid sequence. An asterisk (*) indicates single, fully conserved residues; a colon (:) indicates conservation of strong groups; and a period (.) indicates conservation of weak groups.

The *N*-(deoxy)ribosyltransferase-like superfamily (*N*-ribosyltransferase clan, PFAM entry CL0498) comprises proteins which commonly adopt a flavodoxin-like fold and possess a well-conserved active site architecture. This superfamily encompasses the NDT and ADP-ribosyl cyclase-like families, as well as another family that includes the hypothetical protein PA1492 from *Pseudomonas aeruginosa* with uncharacterized function but known three-dimensional structure (PDB entry 1T1J). Within the NDT family we can distinguish two different subfamilies, i) strict *N*-deoxyribosyltransferases, NDTs (Kaminski, 2002; Fresco-Taboada et al., 2013) and ii) (2′-deoxy)nucleoside-5'-monophosphate *N*-glycosidases, (d)NMP glycosidases (Ghiorghi et al., 2007; Dupouy et al., 2010; Sikowitz et al., 2013; Zhao et al., 2014). Even though both types of enzymes display the canonical “NDT domain” (Pfam PF05014), they possess different substrate specificities. On the one hand, NDTs catalyze the transglycosylation reaction between nucleobases showing a strict specificity for 2′-deoxynucleosides, with very low affinity for ribonucleosides and modified 2′- or 3'-nucleosides (Fernández-Lucas et al., 2011, 2013). On the other hand, (d)NMP glycosidases catalyze the enzymatic hydrolysis of the glycosydic C–N bond in (d)NMPs. However, neither NMP nor dNMP glycosidases have been reported to be active in transglycosylation reactions to date. The different specificity between NMP and dNMP glycosidases is thought to be mostly due to the presence of either a Phe or a Tyr side chain close to the catalytic Glu residue (Sikowitz et al., 2013; Del Arco et al., 2019b) (Figure 1).

Inspired by these reported findings, we recently designed an engineered NDT variant with improved ability to act on ribo- and 2′-deoxyribonucleosides (Del Arco et al., 2019b). Unfortunately, the 2′-deoxynucleoside/ribonucleoside selectivity ratio still indicated a marked preference for 2′-deoxynucleosides. Nonetheless, these findings supported the possible existence of promiscuous wild-type NDTs in Nature.

In our search for atypical NDTs, we found a putative NDT encoded in the *Archaeoglobus veneficus* SNP6 genome (NCBI Reference Sequence: NC_015320.1). The pairwise amino acid sequence alignment against the UniProtKB database revealed that this putative NDT displays the abovementioned canonical “NDT domain” and further BLASTP searches against the Protein Data Bank showed a low sequence identity with a putative NDT from *Enterococcus faecalis* (30%, PDB entry 3EHD) and a type II NDT from *Bacillus psychrosaccharolyticus, Bp*NDT (36%, PDB entry 6EVS) (Fresco-Taboada et al., 2018), with high sequence coverage in both cases (77–97%). The percentage of identity with the type I NDT from *Leishmanhia mexicana* (Crespo et al., 2017) (PDB entry 6QAI) was 41%, but the sequence coverage was below 35%.

Multiple sequence alignment of the putative NDT with well-studied family members revealed that the essential amino acids proposed for nucleoside binding, as well as the catalytic glutamic residue, are conserved in the amino acid sequence of *Av*NRT. These findings strongly support a similar active site architecture. However, the presence of a Phe residue at position 7 instead of the Tyr residue commonly observed in NDTs, suggested to us that this gene might encode a putative intracellular *N*-ribosyltransferase (NRT) that could naturally accept ribonucleosides as substrates (Figure 1).

### Three-Dimensional Model of *Av*NRT

**Figure 2:**
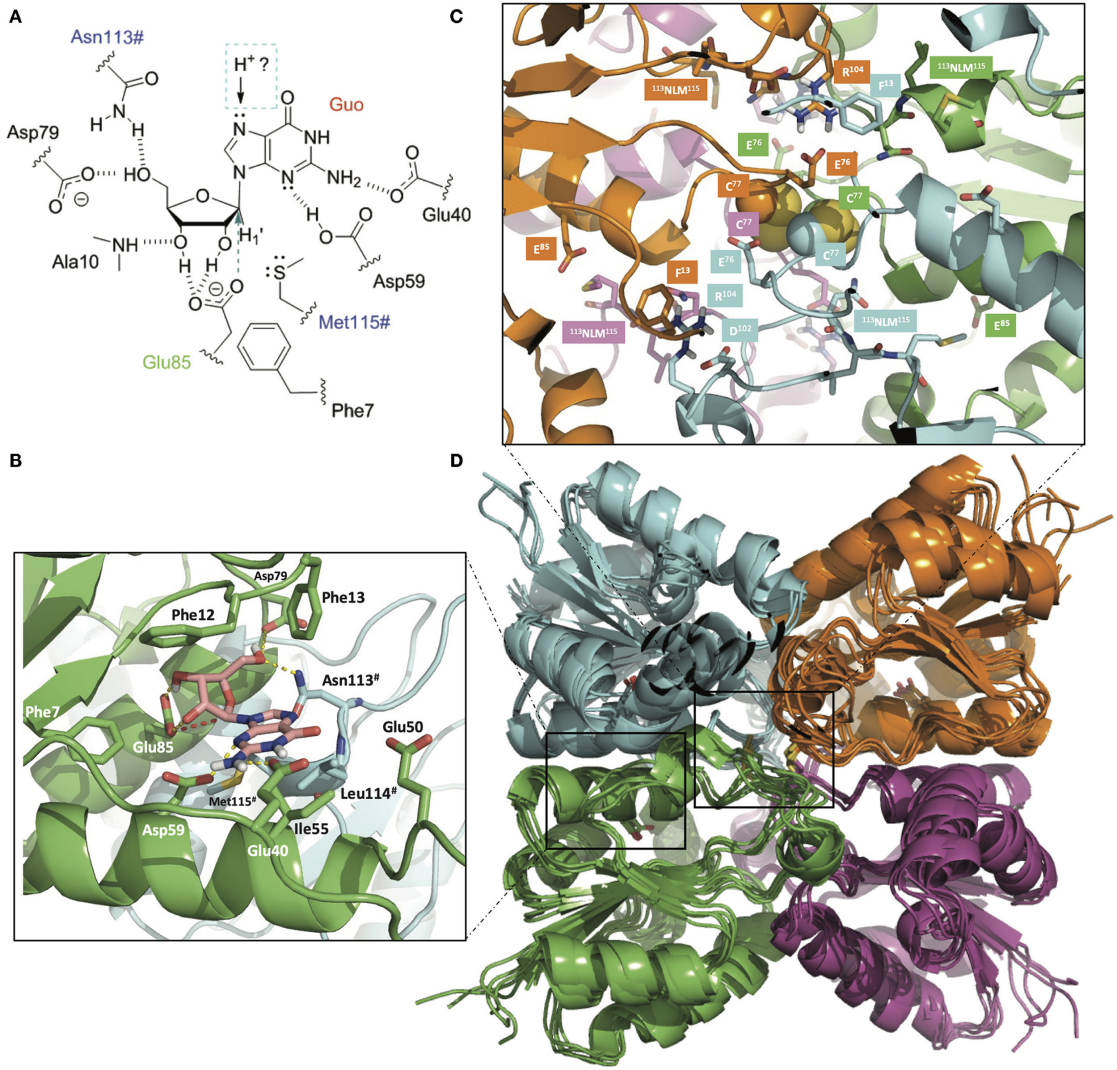
**(A)** Schematic of the binding of guanosine (Guo) to the active site of *Av*NRT. Asn113^#^ and Met115^#^ from the NLM motif (in blue) belong to another monomer making up the obligate dimer that is necessary for nucleobase and sugar recognition. The catalytic glutamate (Glu85) is colored in green and the direction of attack to C1' is indicated by an arrow. The nature of the proton donor to N7, either from the same dimer or from the other dimer making up the tetramer, is presently unknown. **(B)** 3D view of the active site in a homology model of *Av*NRT. Note the hydrogen bonds (dashed yellow lines) that help orienting the carboxylate of the catalytic Glu85 for nucleophilic attack on C1' (red broken line). Enzyme residues relevant for the discussion are shown as sticks and have been labeled. **(C)** Detail of the two disulfide bonds involving Cys77 (C_β_ and S atoms are shown in space-filling representation) putatively found at the core of the tetramer comprising dimers A:B (green:cyan) and dimers C:D (magenta:orange). Note their central positioning and proximity to the four NLM motifs and the catalytic Glu85 (sticks). This arrangement is likely to have a bearing on the catalytic activity due to mutual conformational dependencies of both monomers and dimer of dimers. **(D)** Overlaid ensemble of five *Av*NRT tetramers obtained after the molecular dynamics equilibration of the initial structure and subsequent simulated annealing procedure. Note how the side chain phenyl ring of Phe13 in each monomer provides a suitable flat surface for a stacking interaction with the guanidinium of Arg104 from the equivalent monomer in another dimer, hence contributing to tetramerization. Glu76 and Asp102 are proposed to contribute to dimer and tetramer stabilization by providing a buttressing effect to Arg104. The cartoon representation of the four individually colored monomers and the display of the disulfides as sticks are used for simplicity and ease of visualization. The figure was prepared with PyMOL (Delano, 2002) using ray tracing.

The *Av*NRT protomer is predicted to have the conserved NDT molecular architecture that places the most important residues for nucleoside recognition (from, at least, two subunits) in a suitable orientation for nucleophilic attack on C1' by the carboxylate of Glu85 (Figures 2A,B). Pro11 and Pro42 play a prominent role in making up the active site cavity where one of the Asp79 carboxylate oxygens and the carboxamide nitrogen of Asn113# recognize the O5' of the sugar moiety of the substrate while the backbone NH of Ala10 hydrogen bonds to O3'. The heteroaromatic ring of the nucleobase would thus get sandwiched between the phenyl rings of Phe12 and Phe13, on one side, and the hydrophobic sidechains of Phe56 and Leu119# on the opposite side. The Met120# sidechain sulfur appears close to the H1' atom, which suggests that it likely plays a role, together with the carboxylate of Asp62, in stabilization of the oxocarbenium reaction intermediate, as proposed for other NDTs (Pérez et al., 2018; Del Arco et al., 2019a,b).

We have previously highlighted (Crespo et al., 2017; Del Arco et al., 2018a) that the length of the α3 helix in NDTs is directly related to the enzyme's oligomeric state (~20, ~16, and 14 residues in dimeric, hexameric, and tetrameric enzymes, respectively). Since the residues involved in (A:B-C:D) tetramer stabilization of the putative *Ef* NDT (PDB entry 3EHD) and *Ct*NDT (Del Arco et al., 2018a) are also conserved in *Av*NRT (Figure S1) it seems highly feasible that two obligate *Av*NRT dimers associate into a biologically relevant tetramer (Figures 2C,D). Thus, the ^74^DGECID^79^ loop preceding the catalytic Glu85 would be located at the core of the *Av*NRT tetramer. Besides, the close proximity of the Cys77 side chains strongly suggests the existence of disulfide bridges between C77(A)-C77(B) and C77(C)-C77(D), that is, one cystine per dimer. This tetrameric arrangement, which surely plays a key role in modulating conformational stability and flexibility at high temperatures, is also facilitated by the stacking of the planar guanidinium of Arg104 in the ^101^TDYR^104^ motif onto the phenyl ring of Phe13 in the opposite subunit, *i.e.*, Arg104(A) on Phe13(C), Arg104(B) on Phe13(D), Arg104(C) on Phe13(A), and Arg104(D) on Phe13(B). Stabilization of Arg104 is ensured by buttressing interactions from the carboxylates of Glu76 and Asp102. The side-chain hydroxyl of Thr101, in turn, accepts a hydrogen bond from the NH at the positive end of the ^14^CEAEREFNIKVAEFL^28^ helix dipole.

As regards nucleoside recognition, the presence of Phe7 in a position known to largely dictate the preference for ribonucleosides over 2′-deoxyribonucleosides is indicative of some preference for, or at least tolerance to, the presence of a hydroxyl at the 2′ position of the sugar. In addition, the amino acid composition of the loop region connecting β2 and α3 secondary structure elements serve as an intrinsically disordered active-site flap and is highly variable in the NDT family (i.e., ^39^TDNEATEA^46^ in *Tb*PDT, TDNIATGA in *Lm*PDT, and ^39^QENAAINDKSAYAD^52^ in *Ef* NDT). The equivalent stretch in *Av*NRT is ^43^FISDGSEEEK^58^ and must be responsible for the preferential recognition of nucleosides bearing 2-aminopurines.

### Production and Purification of an *N*-ribosyltransferase From *Archaeoglobus veneficus*

The *nrt* gene, which codes for a putative *N*-ribosyltransferase from the chemolithoautotrophic hyperthermophilic sulfite-reducing archaeal coccus *Archaeoglobus veneficus* SNP6 (*Av*NRT) (Huber et al., 1997), was cloned and overexpressed in *E. coli* BL21(DE3) as described in the experimental section. The recombinant N-terminal His_6_-tagged *Av*NRT was purified by two chromatographic steps. SDS-PAGE analysis of the purified enzyme revealed only one protein band with an apparent molecular mass of 18 kDa (Figure S2).

The sedimentation velocity experiments showed three main peaks with sedimentation coefficients of 1.40 S (*s*_20, w_ = 1.41 S) (7%), 2.24 S (*s*_20, w_ = 2.86 S) (9%) and 4.39 S (*s*_20, w_ = 4.42 S) (84%). The major species found in solution is therefore compatible with a tetrameric state of *Av*NRT (Mw=76.98 kDa) and with a monomer subunit weight of 19.245 kDa, a value similar to that calculated from the amino acid sequence of the His_6_-tagged protein (17.90 kDa).

Different oligomeric states have been described for NDTs from several sources. Thus, *Lactobacillus reuteri* and *L. helveticus* (Kaminski, 2002; Fernández-Lucas et al., 2010) NDTs have been shown to be hexameric, *Chroococcidiopsis thermalis* (Del Arco et al., 2018a) and *Lactococcus lactis* (Miyamoto et al., 2007) NDTs are tetrameric, and the PDTs from the protozoans *Trypanosoma brucei* and *Leishmania mexicana* (Crespo et al., 2017; Pérez et al., 2018) are dimeric.

To assess the hydrolytic activity of *Av*NRT, that is, the first step of the transglycosylation reaction, the enzymatic hydrolysis of different ribo- and deoxyribonucleosides was performed. As shown in Table S1, *Av*NRT displays low activity values in all cases, ranging from 0.002 to 0.02 IU/mg, with a remarkable preference for 2-amino and 2-halopurine nucleosides. Strikingly, no hydrolytic activity was observed on pyrimidine nucleosides. On the basis of these results, the enzymatic hydrolysis of Guo was chosen as the standard reaction for further experiments.

### Temperature and pH Dependence of *Av*NRT Activity

**Figure 3:**
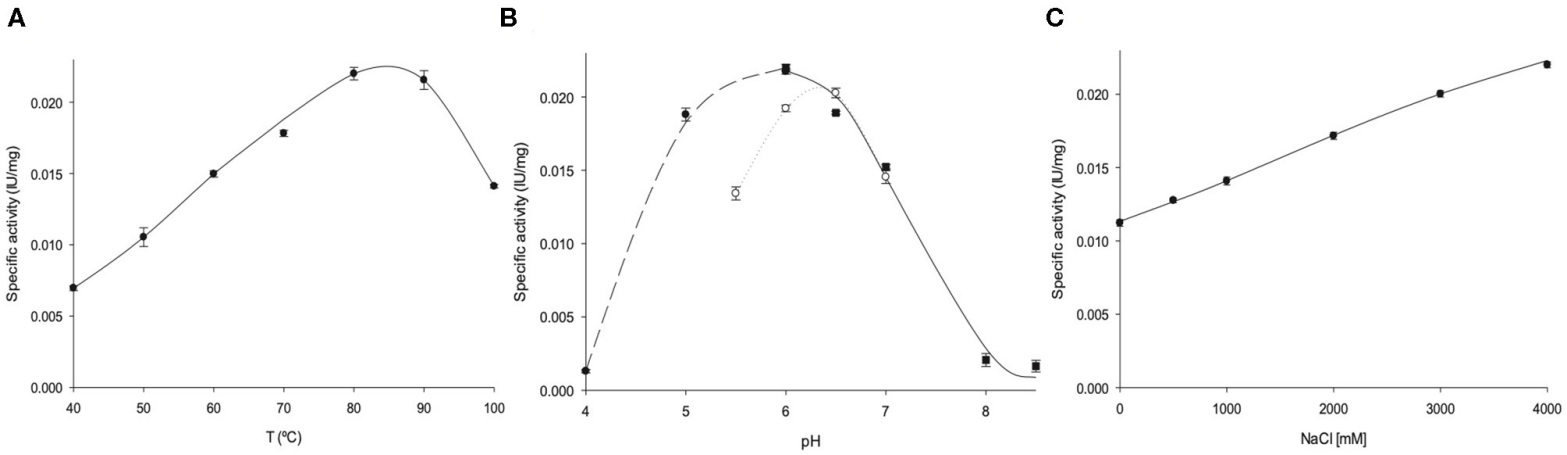
Biochemical characterization of *Av*NRT activity. **(A)** Temperature dependence. **(B)** Effect of pH, (•) sodium citrate 50 mM (pH 4–6), (◦) MES 50 mM (pH 5.5–7), (■) sodium phosphate 50 mM (pH 6–8.5). **(C)** Effect of ionic strength.

As expected for a hyperthermophilic enzyme, the activity of *Av*NRT was found to be largely preserved (≥ 60%) at high temperatures (60–100°C), with a peak between 80 and 90°C (Figure 3A). These results agree with those reported for hyperthermophilic *Ct*NDT (80% of the maximum activity at 70–90°C), the only precedent of a thermophilic NDT (Del Arco et al., 2018a). Moreover, as described more exhaustively below, several disulfide bonds are present in *Av*NRT which also reinforces these experimental results. The pH profile reveals that *Av*NRT activity is largely preserved (≥60%) across the pH range 5–7 with a peak at pH 6 (Figure 3B). However, as shown in Figure 3B, the enzymatic activity is strongly affected by the nature of the solution buffer.

### Effect of Ionic Strength on *Av*NRT Activity

*Archaeoglobus veneficus* SNP6 reportedly grows in a NaCl range 0.5–4% (optimum 2%) (Huber et al., 1997). In this respect, this coccus can be considered as a moderate halophile (optimal growth between 3 and 15% NaCl) (De Lourdes Moreno et al., 2013) and therefore only a mild tolerance for medium salt concentrations was expected for *Av*NRT. Surprisingly, the enzyme shows a remarkable salt dependence and a 2-fold increase in enzymatic activity was observed at high salt concentrations (4M NaCl) (Figure 3C). This unusually high halotolerance is more common for enzymes from extreme halophiles (optimal growth between 15 and 30% NaCl).

Halophilic proteins usually display a high number of negatively charged, surface-exposed amino acids (Asp and Glu). This characteristic, together with a decrease in the content of basic (Lys and Arg) and hydrophobic residues, leads to a highly negative molecular electrostatic potential (MEP) surface which is likely to enhance interactions with the surrounding environment and promote protein stability at high salt concentrations (Madern et al., 2000; Oren and Mana, 2002; Sinha and Khare, 2014). This enrichment in acidic residues on haloenzymes has been attributed to an “evolutionary adaptation” of halophiles that enabled their proteins to adapt under saline stress and suitably alter inherent protein-solvent interactions. In this respect, negatively charged surface patches bind hydrated ions to form a solvation shell that protects the enzyme from aggregation under high salinity conditions. Another proposed hypothesis to explain this effect is the presence in halophilic proteins of a great number of salt bridges, which provide a higher structural rigidity. In the absence of high salt concentrations, the ordered water molecule network on the protein surface is lost, and the water molecules interfere with salt bridge formation (Madern et al., 2000; Oren and Mana, 2002; Sinha and Khare, 2014).

**Figure 4:**
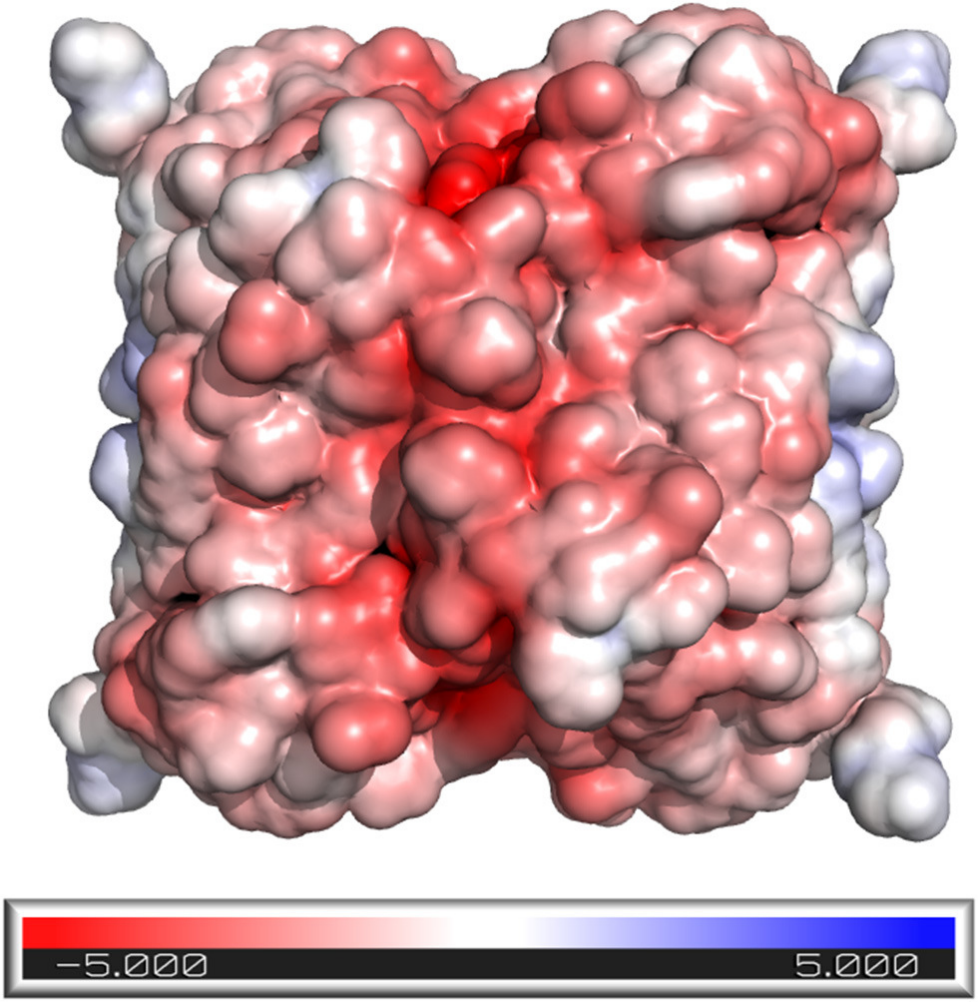
*Av*NRT surface colored according to the molecular electrostatic potential [ranging from −5.0 (red) to 5.0 (blue)] computed using the APBS program (Jurrus et al., 2018), as implemented in PyMOL (Delano, 2002).

The *in silico* analysis of the amino acid sequence showed that *Av*NRT displays a higher number of acidic residues (17.5%) than other reported NDTs (calculated from published data), such as *Tb*PDT (14.3%) (Pérez et al., 2018), *Lm*PDT (12.9%) (Crespo et al., 2017), *Ld*NDT (15.9%, Acosta et al., 2018), *Lr*NDT (15%) (Fernández-Lucas et al., 2010) or *Bp*DNT (15.5%) (Fresco-Taboada et al., 2018). Additionally, most of these acidic residues are surface-exposed (88%) and strategically located in several clusters distributed over the protein surface so as to provide a highly negative surface MEP (Figure 4). These features are in line with the high halodependence exhibited by *Av*NRT.

Since neither NDTs nor PDTs have shown this extreme salt tolerance (Fernández-Lucas et al., 2010, Crespo et al., 2017; Acosta et al., 2018; Fresco-Taboada et al., 2018; Pérez et al., 2018), *Av*NRT emerges as the first halotolerant NDT and this sole fact can make it an interesting biocatalyst for industrial applications.

### Thermal Stability

**Figure 5:**
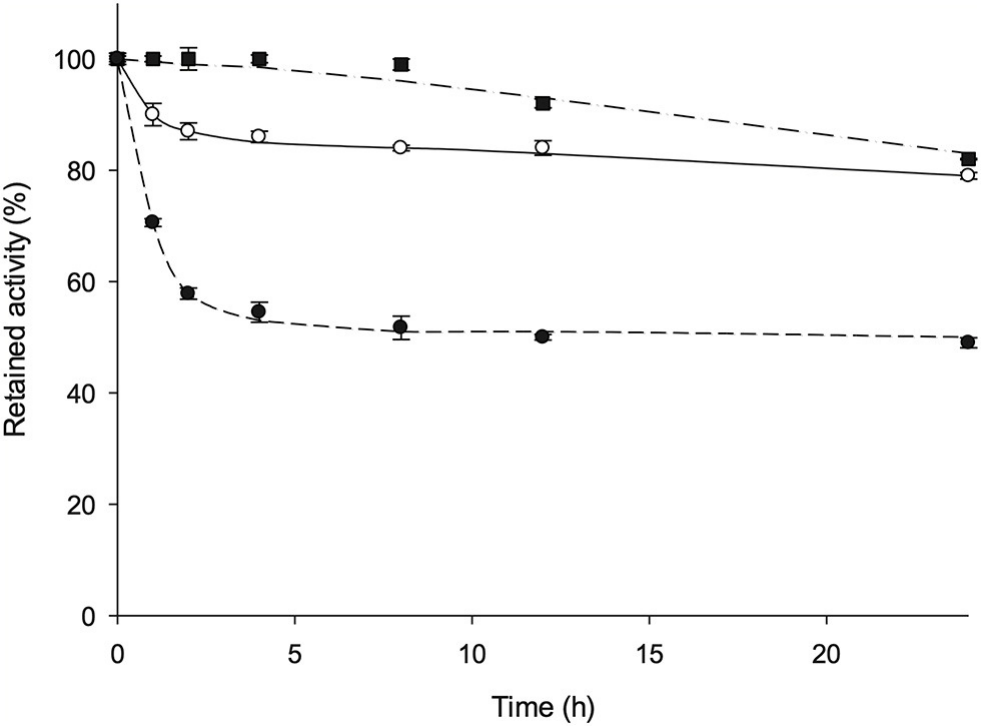
Time course of the thermal inactivation of *Av*NRT at (■) 60°C, (◦) 70°C, and (•) 80°C in 10 mM sodium phosphate pH 7.

*Av*NRT activity does not decline when the enzyme is stored at 4 and−80°C in 10 mM sodium phosphate buffer, pH 7 for 365 days. Furthermore, the effect of temperature on enzyme stability was evaluated by incubating *Av*NRT over a temperature range 60–80°C in 10 mM sodium phosphate buffer, pH 7. Figure 5 shows that *Av*NRT did not suffer any loss of activity after being maintained at 60°C for 8 h. Moreover, the activity was not significantly decreased after incubation periods longer than 20 h (79–82% retained activity) at 60 and 70°C. Interestingly, *Av*NRT showed a considerably high half-life (around 8 h) when stored at 80°C, which was not unexpected given the origin of this enzyme.

In addition, our *in silico* homology model revealed the high likelihood of one intradimeric disulfide bond between the spatially very close Cys77 and Cys77# of two monomers. Therefore, the putative presence of two disulfide bridges in the tetramer would lead to a more compact structure and restrained protein mobility (Figures 2C,D) and this can account for the high thermostability displayed by *Av*NRT. This hypothesis is supported by the fact that *Av*NRT suffers a drastic loss of activity (around 90%) over the course of 1 h upon incubation at 80°C for 2 h (Figure S3) when incubated in presence of 3 mM DTT (disulfide reducing agent).

### Effect of Water-Miscible Solvents on *Av*NRT Activity

Haloadaptation is perceived as a protective mechanism that confers stability to halophilic proteins against denaturants such as high temperature or the presence of chaotropic agents and/or organic solvents. Water-miscible solvents disrupt hydrogen bonding interactions among protein subunits and also reduce water concentration at the active site, thus reducing catalytic efficiency (*k*_*cat*_). Due to the fact that high salt concentrations decrease water activity significantly, enzymes from halophilic microorganisms often work in low-water media and remain properly folded and active in the presence of organic solvents (Sinha and Khare, 2014; DasSarma and DasSarma, 2015). In this respect, the protective role of high salt concentrations against the disruptive effect of organic solvents is extensively reported in the literature (Madern et al., 2000; Marhuenda-Egea and Bonete, 2002; Sinha and Khare, 2014).

**Figure 6:**
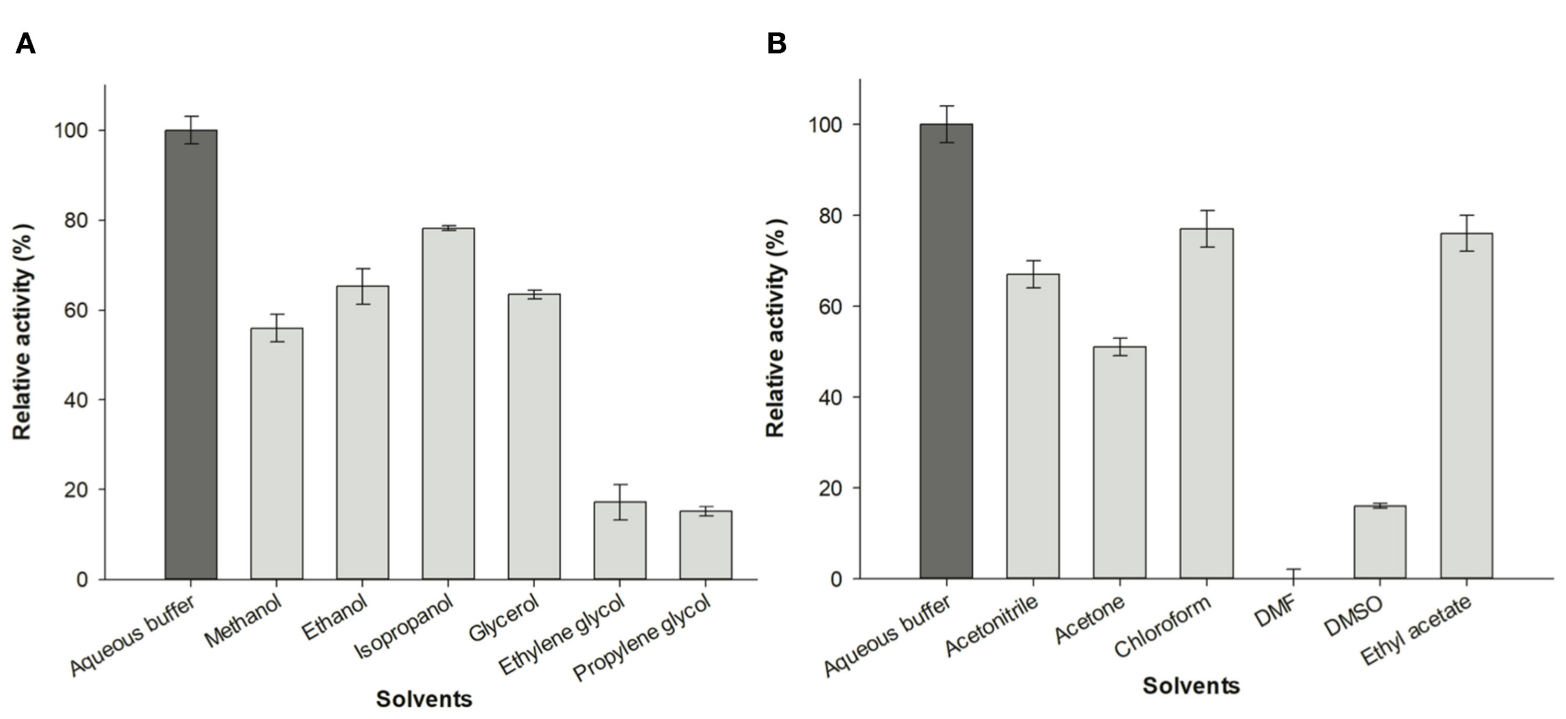
Effect of organic co-solvents (20% v/v) on enzymatic activity of *Av*NRT. **(A)** Alcohols and polyols. **(B)** Aprotic polar solvents.

To investigate the effect of water-miscible solvents on the activity of *Av*NRT, the enzymatic hydrolysis of Guo was assayed in the presence of 20% (v/v) polar protic (MeOH, EtOH, isopropanol, glycerol, ethylene glycol, and propylene glycol) and aprotic co-solvents (acetonitrile, acetone, chloroform, *N, N*-dimethylformamide, DMSO and ethyl acetate). As shown in Figure 6, a general reduction of enzyme activity in the presence of the solvents tested can be observed. However, this activity decrease is very different depending upon the nature of co-solvent. On the one hand, we can observe a different behavior between short-chain alcohols and polyols. Thus, the short chain alcohols displayed higher relative activities (52–73%) than polyols (15–17%), being glycerol the only exception (56%) to this tendency (Figure 6A). In this respect, the size and/or conformation of polyols clearly has an intrinsic effect on enzyme activity. Thus, an increase of enzymatic activity is apparent as the dielectric constant (ε) decreases, in the following order: isopropanol (72% relative activity, ε = 20, log *P* = 0.05) > EtOH (65% relative activity, ε = 25, log *P* = −0.24) > MeOH (65% relative activity, ε = 33; log *P* = −0.76); in contrast, the enzymatic activity increases as the log *P* value of the short-chain alcohols increases (Fernández-Lucas et al., 2012; Del Arco et al., 2018a). On the other hand, we can observe an increase of enzyme activity linked to the increase of log *P* value for aprotic co-solvents (Figure 6B), with maximum relative activity for ethyl acetate (76%, log *P* = 0.73) and a high or practically total loss of activity in the presence of DMSO (16% relative activity, log *P* = −1.3) and *N, N*-dimethylformamide (no activity, log *P* = −1.0). In addition, this log *P* dependence was also conserved using chloroform, a non-water-miscible solvent (77% relative activity, log *P* = 1.83) (Fernández-Lucas et al., 2012; Del Arco et al., 2018a).

### Enzymatic Synthesis of Purine Nucleoside Analogs

With a view to the possible industrial implementation of *Av*NRT as a biocatalyst for the synthesis of nucleoside analogs, we assayed the transglycosylation ability of *Av*NRT on different purine nucleobases (2-FAde, 2-ClAde, 2,6-DAP, 6-MeOGua, Ade and Hyp) using Guo or dGuo as the nucleoside donors (Table 1). No transglycosylation activity was observed when dGuo was used as the donor (data not shown) and the transglycosylation seemed to be strongly linked to the presence of an amino group on C2 of the purine ring (Table 1, Figure 2B). Thus, low activity values were obtained for Ade and 2-halopurines and no activity whatsoever was observed when Hyp was used as the acceptor base. A putative interaction between the 2-amino group and the carboxylate of Glu40 (Figure 2B) could account for these striking differences.

**Table 1:** Glycosidic bond cleavage in guanosine by *Av*NRT and ribosyl transfer to different heteroaromatic bases used as acceptors^a^.

**Donor**	**Acceptor**	**Time (min)**	**Released gua (%)**	**Nucleoside conversion (%)**
Guanosine	2-ClAde	15 45 120	19 ± 0.5 45 ± 2 48 ± 1	0.5 ± 0.2 6.5 ± 0.5 7 ± 1
	2-FAde	15 45 120	21 ± 1 48 ± 0.5 52 ± 1.5	2.5 ± 0.5 5 ± 2 8 ± 1
	2,6-DAP	15 45 120	29 ± 1 50 ± 1 49 ± 2	7 ± 0.6 38 ± 2.0 41 ± 1.0
	6-MeoGua	15 45 120	24 ± 0.5 48 ± 2 62 ± 1	16 ± 1 34 ± 1 40 ± 1
	Ade	15 45 120	19 ± 2 45 ± 2 48 ± 1	0.5 ± 0.1 8 ± 1 8 ± 0.5

a *Reaction conditions: 22.4 μg of enzyme in 40 μL at 80°C, 20 min. [Substrates] = 0.75 mM, in 50 mM sodium phosphate buffer, pH 6*.

These results agree with the hypothesis derived from the bioinformatic analysis (Figures 1, 2B) suggesting a strict preference of *Av*NRT for ribonucleosides. Furthermore, they are also in line with those previously reported for *Tb*PDT demonstrating that the Tyr5 → Phe replacement enhances the ability of this enzyme to accept ribonucleosides as substrates (Del Arco et al., 2019b).

*Av*NRT is unusual insofar as it cannot be strictly classified as a type I or type II NDT and should therefore be considered as part of a novel subfamily, type III, which only acts on ribonucleosides (NRTs). Moreover, the greater activity of *Av*NRT on 2-purine ribonucleosides suggests the possibility that *Av*NRT may have originated from a type I (PDT) or *vice versa*. In light of the results presented here, there appears to be a growing need for a re-classification of NDTs.

## Conclusions

A novel *N*-ribosyltransferase from *Archaeoglobus veneficus* (*Av*NRT) is reported as the first wild-type member of the *N*-(deoxy)ribosyltransferase-like superfamily that acts selectively on ribonucleosides. Experimental evidence revealed *Av*NRT to be a tetramer, as expected from previous bioinformatic analysis and molecular modeling results. *Av*NRT is active and stable at high temperatures (60–80°C) and displays optimal activity at pH 6 and 4 M NaCl. This novel halotolerant and thermostable catalyst was employed in the enzymatic synthesis of different purine ribonucleoside analogs. *Av*NRT expands the versatility of previously reported NDTs and poses new questions about the biological significance and evolution of NDTs.

## Data Availability Statement

The original contributions presented in the study are included in the article/Supplementary Material, further inquiries can be directed to the corresponding author.

## Author Contributions

JF-L and FG are mainly responsible for the experimental design and research coordination. JA, JD, and VP contributed to the development and analysis of experimental data. All authors contributed to the article and approved the submitted version.

## Conflict of Interest

The authors declare that the research was conducted in the absence of any commercial or financial relationships that could be construed as a potential conflict of interest.
