# Supplementary material for: N-Ribosyltransferase From Archaeoglobus veneficus: A Novel Halotolerant and Thermostable Biocatalyst for the Synthesis of Purine Ribonucleoside Analogs
Source: Front Bioeng Biotechnol. 2020 Jun 16;8:593. doi: 10.3389/fbioe.2020.00593 (PMC7308715; doi:10.3389/fbioe.2020.00593)
Supplement: Supplementary file 1 [file Data_Sheet_1.PDF]

Frontiers in Bioengineering and Biotechnology

N-ribosyltransferase from *Archaeoglobus veneficus*: a novel halotolerant and thermostable biocatalyst for the synthesis of purine ribonucleoside analogues.

Javier Acosta [1+], Jon Del Arco[1+], Víctor Pisabarro[1], Federico Gago[2], Jesús Fernández-Lucas *[1,3]

[1]Applied Biotechnology Group, Universidad Europea de Madrid, Urbanización El Bosque, E-28670 Villaviciosa de Odón, Madrid (Spain).

[2]Department of Biomedical Sciences and "IQM-CSIC Associated Unit". School of Medicine and Health Sciences, University of Alcalá, E-28805 Alcalá de Henares, Madrid (Spain)

[3]Grupo de Investigación en Ciencias Naturales y Exactas, GICNEX, Universidad de la Costa, CUC, Calle 58 # 55 – 66, Barranquilla (Colombia).

***Correspondence:**
Jesús Fernández-Lucas
E-mail: jesus.fernandez2@universidadeuropea.es

Electronic Supplementary Information

```
AvNRT     MAGLK-VFLAAP-FFCEAEREFNIKVAEF--LRDNGFE-VWMAQENPFISDGSEEEK-----RRI    55
CtNDT      MKRKIIYLASPYGFSQQQKTLLLPPI----VRALEALGIEVWE--PFARNNQIDFSQADWAYRV    48
EfNDT        MTKIYFAGP-LFSQAD-LRYNA-YLVEQIRQLDK---TIDLYLPQENAAINDKSAYADSKMI    46
TbPDT        MRKIYIAGPAVFNPDMGASYY-----NKVRELLK-KENVMPLIPTDNEATEALDI-------    43
LmPDT     MPAPKTIYIAGPAVFHPDNGEAYY-----NNVRALMKGK-DVVPLIPTDNIATGAVNI-------    46
                   * *  *                 *             *

                                          77
AvNRT     FEMDLSALKGCDAVVAVLDG---ECIDSGTAFELGYAYAMGKPIIGIKTDYRTFSSIE-------   110
CtNDT     AQADLQDVKNCDGIFAVVNG---TPPDEGVMVELGMAIALNKAIFLFRDDFRRCSDNERY-----   104
EfNDT     ALADTENVLASDLLVALLDG---PTIDAGVASEIGVAYAKGIPVVALYTDSRQqgadnhqkldal   102
TbPDT     RQKNIQMIKDCDAVIADLSPfrgHEPDCGTAFEVGCAAALNKMVLTFTSDRRNMR--EKYgsgvd    98
LmPDT     RNKNIDMIRACDAIIADLSPfrsKEPDCGTAFELGYAAALGKVLLTFSTDTRPMV--EKYGSEMA   101
                     *   *          * *   *:*                *

AvNRT     -----------GLNLMIEVAVrlikastFEElkgrllealrdvkp                      144
CtNDT     -----------PLNLMLFAGLpeigwenyyytsvdeiqshdkalykwltgm                155
EfNDT     neiae-NQFH-YLNLYTV-GLiklngrvvsseedlleeikqrls                       159
TbPDT     kdnLRVEGFGLPFNLMLYDGV--evfdsFESafkyflanfpsk                        152
LmPDT     DG-LSVENFGLPFNLMLHDGT--dvfdsFEAafayfvehhltp                        155
                       **
```

Figure S1. Structure-based multiple sequence alignment of experimentally determined NDTs from *Enterococcus faecalis* (*Ef*NDT, PDB id. 3EHD), *Trypanosoma brucei* (*Tb*PDT, PDB id. 2A0K), and *Leishmania mexicana* (*Lm*PDT, PDB id. 6QAI) used as templates for homology modeling of *Av*NRT and *Ct*NDT, whose three-dimensional structures are presently unknown. Strictly conserved residues are marked with an asterisk and those positions relevant to the discussion have been highlighted and/or colored.

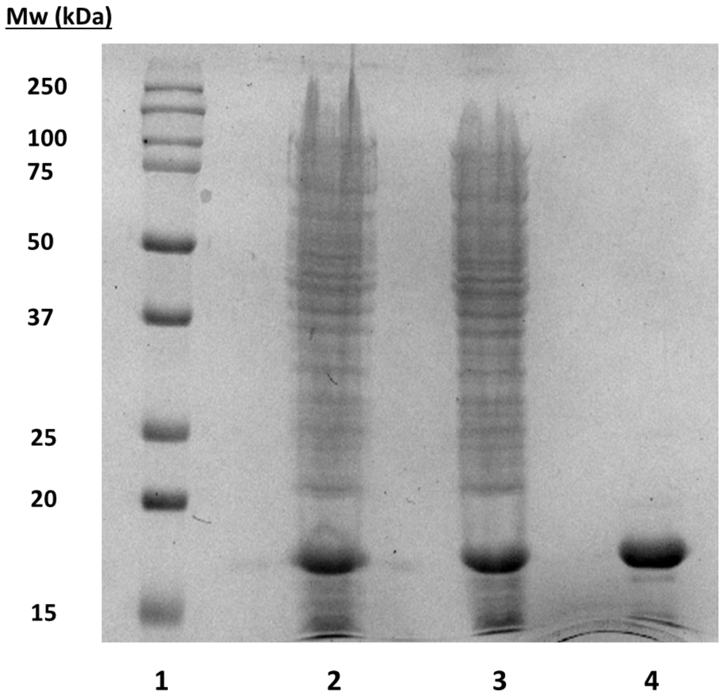

Figure S2. SDS-PAGE analysis of soluble *Av*NRT. Lane 1. Precision Plus Protein™ prestained standard from BioRad used as a molecular weight marker. Lanes 2 and 3. Supernatant obtained after centrifugation of the lysed cells. Lane 4. Sample (18 µg protein) after purification.

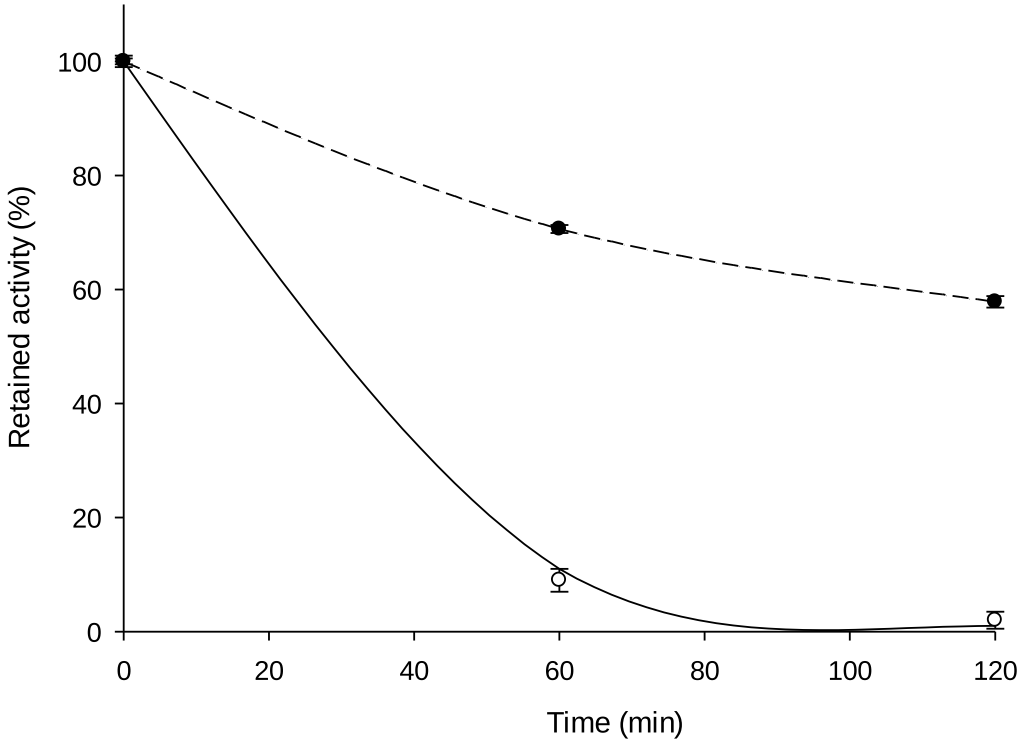

Figure S3. Time course of the thermal inactivation of *Av*NRT in 10 mM sodium phosphate pH 7 at 80 °C in the absence (•) or presence (○) of 3 mM DTT.

Tables

Table SI. Enzymatic hydrolysis of ribo- and 2'-deoxynucleosides catalyzed by *Av*NRT[a]

**Nucleoside**	**Nucleobase release (%)**	**Specific activity (IU/mg)**
**Ado**	2.5	0.002 ± 0.0005
**Guo**	25	0.022 ± 0.0021
**Ino**	2.4	0.002 ± 0.0009
**Urd**	n.d.	n.d.
**dAdo**	1.3	0.001 ± 0.0007
**dFAdo**	12.6	0.011 ± 0.0003
**dClAdo**	24	0.021 ± 0.0034
**dCyd**	n.d.	n.d.
**dGuo**	24	0.021 ± 0.0003
**dIno**	1.3	0.001 ± 0.0009
**dThd**	n.d.	n.d.
**dUrd**	n.d.	n.d.

[a] Reaction conditions: 22.4 µg of enzyme in 40 µL at 80 ºC, 20 min. [Substrates] = 1 mM, 50 mM sodium phosphate buffer, pH 6. n.d.: not detected.
